# Digital technologies for social inclusion of individuals with disabilities

**DOI:** 10.1007/s12553-018-0239-1

**Published:** 2018-06-24

**Authors:** Mirfa Manzoor, Vivian Vimarlund

**Affiliations:** 1 Department of Informatics, Jönköping International Business School, Jönköping University, Jönköping, Sweden; 2 Department of Computer Science, Linköping University, Linköping, Sweden

**Keywords:** Digital technologies and services, Disabilities, Social inclusion

## Abstract

Information technology can be an important facilitator of social inclusion for people with disabilities into society. However, the goals specified in this area by organizations such as the European Commission have not yet been achieved in their totality. The aim of this paper is to explore which types of information communication technology-based applications and/or digital services have been suggested to facilitate the social integration of people who suffer from different types of disabilities. We performed a literature review that included studies published during a period of 6 years (2010–2016). The results show that, in the data we have had access to, no concrete patterns can be identified regarding the type of technology or technological trends that can be used to support the social integration of individuals with disabilities. This literature review is of relevance to the identification of further research areas and to the identification of issues which have to be considered in the context of the development and implementation of technological innovations that are aimed at promoting or facilitating social inclusion of individuals with disabilities.

## Introduction

People with disabilities^1^ face a number of challenges in today’s society [1–3]. According to the European Commission, the overall employment rate of people with disabilities in Europe is 48%. Only 27.8% of people with a disability obtain a tertiary level degree or diploma [4], and approximately 70% of people with disabilities face poverty or issues that are related to social inclusion [5]. The absence of effective support services, for example, that allow for or facilitate access to transportation, building access, access to information and communication in different formats and through different platforms and systems results in a situation where people with disabilities are forced to rely on their families, something which, we claim, prevents them from being socially included and integrated into society [6].

Digital technology has been described as a facilitator for social inclusion, because it allows for the delivery of real-time services that can enable individuals to learn, work, travel, socialize, shop, and interact with the community without being subject to physical barriers [7, 8]. Digital technologies have also been identified as one of the most important factors that can contribute to reducing existing social gaps and can be used to encourage and support social inclusion and increase people’s quality of life [9]. However, for different reasons, the implementation of IT in this specific area has not yet been fully realised. Consequently, technology-based applications are still not used as generic enablers in the promotion of social inclusion for people with disabilities.

According to National Council Disability [10], “the more reliant society becomes on technology to perform fundamental aspects of every-day living, how we work, communicate, learn, shop, and interact with our environment, the more imperative it is that people with disabilities have access to that same technology, and the more costly will be the consequences of failure to ensure access”. Although there are a number of different information technologies that have been used to develop applications for disabled people [11], only a few empirical studies have been conducted into examining the technology trends used to develop applications for disabled people.

Some research studies [9–16] indicate that, despite the number of emerging technologies that have been suggested to support individuals with disabilities, the expected goals with respect to the integration of individuals with disabilities into society have not been achieved. We also note that there is but a limited number of studies that discuss technologies that are aimed at supporting the development of applications that focus on how social inclusion for people with disabilities can be facilitated [11–16].

The aim of this paper is to explore the various ICT-based applications and/or digital services that have been suggested to support individuals with disabilities in their integration into society^2^ [17].

## Method

A literature review was performed covering a period of time of 6 years (July 2010 to November 2016) with a focus on studies that describe either the design or the development of ICT-based applications and web-based services for people with disabilities. The International Classification System of Functioning, Disability and Health (ICF) [18] definition was used to search for and select the studies that were included in this literature review.

**Fig. 1:**
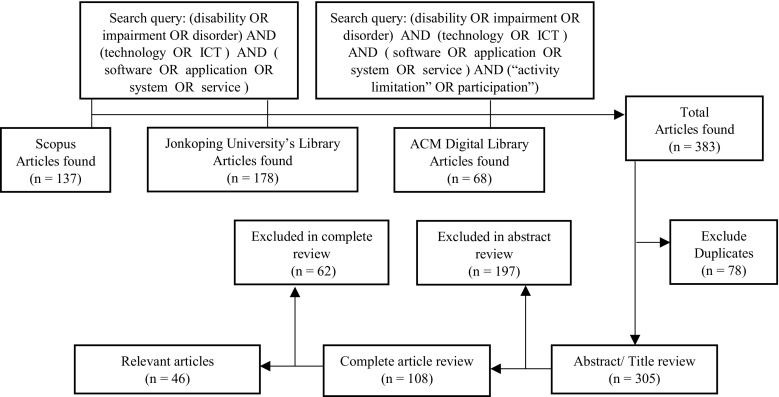
The search process and the number of articles found

The search for the relevant publications was conducted by using databases such as Scopus, the ACM digital library, and Jonkoping University’s library which has access to a number of databases, including Science Direct, Elsevier, Sage, the Wiley Online library, Taylor & Francis Online, and Springer. The search process included several steps. At the first step, we collected articles that included the search terms (see Fig. 1, below) in the title, abstract, or in the keywords of the sample. We also searched for publications that used alternative terms such as *impairment*, *special needs*, *software*, *system*, and *services*. We limited our search to articles that were published in scientific journals, in English. Books, abstracts, and studies published in non-peer-reviewed journals were not considered. The search process is illustrated in Fig. 1.

In total, we found 383 articles that contained one or more of the search terms. After removing 78 duplicated articles, 305 articles were selected in the first round. Further 197 articles were excluded because: (i) they did not present a discussion of how ICT- applications or digital services can support disabled individuals in their social integration, (ii) they focused on assistive devices, such as power wheelchair, robots, electric canes, for example, (iii) they focused on how diagnosis, treatment, and rehabilitation programs can be improved with the use of ICT (iv) they focused on how policies should change in response to the use of ICT-based applications. Articles that presented discussions of applications or web-based services that can be used by therapists, care givers, and physicians in their work with disabled individuals were also excluded from the sample. The reason why these articles were excluded was they did not have the stated aim of facilitating the social inclusion of disabled *individuals*. Instead, they described how certain technologies can be used to facilitate interaction between experts and patients, or to support experts in their daily work. The fact that the notion of ‘social inclusion’ was not addressed in these articles was the reason for their exclusion from the literature review set.

In the next step, articles that discussed or focused on issues such as the implementation, design, and development of technology-based applications for disabled individuals with the aim to support them in their daily routines or to support them in their interaction with society were included. A total of 108 articles were read their totality. Out of these 108 articles, an additional 62 articles were excluded after a second review because they discussed the benefits of assistive devices for smart homes in general, but they did not mention or suggest applications that could be used to support individuals with any kind of disability.

The data included in this paper consists of 46 articles that include a discussion of technologies that can be used to support disabled individuals with their inclusion into society. From the selected articles, the following information was extracted: (i) publication year, (ii) study objective, (iii) the technology discussed in the study, (iv) the type of application(s) or service(s), (v) the functionality of the application(s), and (vi) the type of disability they aim to interact with. (See Appendix 1.)

## Results

This section presents the results of the literature review described in the previous section. The results show (i) the number of articles included in the review, year by year, (ii) the type of technologies that were discussed in the articles, and (iii) the different types of disability that the technological solutions aimed to support.

### The number of studies that described or discussed technologies that can be used to support different types of disabilities

**Fig. 2:**
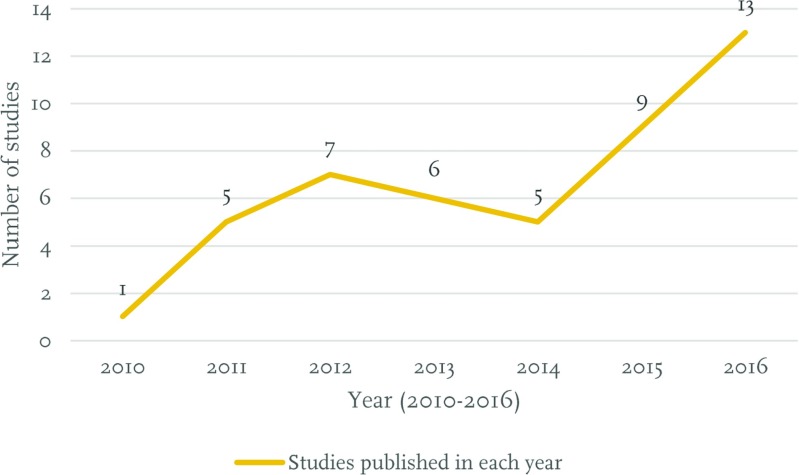
The aggregate number of articles included in the literature review in terms of their year of publication

Below, Fig. 2 shows the distribution of the 46 selected studies in terms of their year of publication.

From the table above, we can see that no specific pattern or year-by-year systematic increase in the number of publications that were included in our literature review data set was identified. However, what is clear is that the number of published studies has increased during 2015 and 2016.

### The types of technologies that were discussed in the articles

Different terms were used in the various articles to discuss or describe technologies that can be used support different types of disabilities over the time. A list of the terms that were used is presented in Table 1.

**Table 1:** The terms used to describe technologies as enablers

Year	The technological terms used in the selected articles
2010	communication technology
2011	adaptive technology, assistive technology, instructional technology, web technology
2012	communication technology, assistive technology, web technology, tele-rehabilitation technology, internet-based technology
2013	communication technology, assistive technology, instructional technology, mobile technology, speech recognition technology, gerontechnology
2014	assistive technology, instructional technology, virtual technology, telecommunication technology, electronic technology, access technology
2015	assistive technology, tele-rehabilitation technology, telecommunication technology, social media technology, wearable technology, mobile technology, information technology
2016	assistive technology, tele-rehabilitation technology, mobile technology, virtual technology, GPS technology, screen reader technology, game technology, voice recognition technology, 3D printing technology

The results shown that the study published in 2010 [1] used the general term such as *communication technology* to develop services for people with disabilities.

The studies published in 2011 have used more terms as compared to studies published in 2010 such as: *adaptive technology* [2], *assistive technology* [3, 4], *instructional technology* [5], and *web technology* [6]. The terms *web technology* and *instructional technology* were kept as general terms. Moreover, the terms *adaptive technology* that has made a thoughtful change in attitude and technology [7] was also used in the mentioned year. It is important to mention here that the terms ‘adaptive technology’ and ‘assistive technology’ were often used interchangeably and the adaptive technology considered as a subset of assistive technology [7].

The studies published during the year 2012 used some new terms to design and develop services such as: tele-*rehabilitation technology* [8] and *internet-based technology* [9, 10]. Apart from this, some already used terms during the year 2010–2011 have also been discussed in 2012. For instance, *communication technology* [11], *assistive technology* [11, 12], *web technology* [13, 14]*.*

The studies published in 2013 shown advancement in the technology because a number of new terms discussed during this year such as: [15], *mobile technology* [16], *speech-recognition technology* [16], and *gerontechnology* [17]. It has observed that the studies published during this year also discussed sustainability of aging society and thus used the term *Gerontechnology* to provide good health, independent living and full social participation up to a high age [18]. Some other terms such as *communication technology* [19, 20], *assistive technology* [16, 21], *instructional technology* [15] also used in these studies.

Virtual technology [22] is another new term that was used in studies published during the year 2014 for enabling physical activity with the aim to improve impairments, activity limitations, or participation. We also found a term *assistive technology* [23–25] with the focus to enhance physical activities of people with disabilities. Some general terms such as: *telecommunication* [26], *electronic technology* [23], and *access technology* [24] were also discussed in studies published in 2014.

Studies published during 2015 continued to use terms such as *assistive technology* [27–30], *tele-rehabilitation technology* [31, 32], *telecommunication technology* and added new terms such as *social media technology* [28], *wearable healthcare technology* [33], *mobile technology* [27] whilst general terms, such as *technology* [34] and *information technology* [35] were kept as general terms.

Studies published in 2016 used terms such as *assistive technology* [36–40], *tele-rehabilitation technology*, *Mobile technology* [37], *virtual technology* [41], *GPS technology* [42, 43], *screen reader technology* [36], and *rehabilitation technology* [44, 45]. During 2016, the articles also included terms not previously used, including *game technology* [46], *voice recognition technology* [38, 39], and *3D printing technology* [47].

A common term that was used across most of the time period under examination (from 2011 to 2016) is *assistive technology*. There is, however, no specific definition of what *assistive technologies* consist of in the selected studies. Other terms that were used in the various articles seem to follow some technological trends and innovations to support people with disabilities [55, 57–59, 63]. Further details on technologies can be seen in the appendix.

### The various types of disability that were discussed in the selected articles

A number of different types of disability were discussed in the articles included in our literature review. In Table 2 below, the number of articles and the type of disability they discuss are presented.

**Table 2:** Number of studies published each year with focus on different types of disability AND elderly

Year	Cognitive disability	Physical disability	Visual impairment	Intellectual disability	Elderly	Parkinson’s disease	Disability in general	Total studies
2010	1	0	0	0	0	0	0	1
2011	0	2	1	0	0	0	2	5
2012	2	2	2	0	0	1	0	7
2013	1	1	0	2	2	0	0	6
2014	2	0	0	1	1	1	0	5
2015	1	3	0	2	1	1	1	9
2016	4	1	1	3	1	1	2	13

The results presented in Table 2, shown that in 2010, one (1) publication focused on the area of *cognitive disability* [19]. The purpose for concentrating on this area was to keep people with cognitive disability, healthy and increasing their engagement in the online health care system (see appendix). The studies published during the year 2011 had a different focus than studies published in 2010. The studies published in 2011, focused on *physical disability* [20, 21], *visual impairment* [22], or *disability in general* with no specific definition of the kind of disability referred to [23, 24]. However, the studies published in 2012, only added one new type of disability (*Parkinson’s disease* [30]) that had not been discussed before in studies published during the year 2010 and 2011.

The studies published in 2013, have broadened the focus and added more disabilities to discuss in the studies that include *physical disability* [34], *cognitive disability* [32, 36], and *elderly* [33, 37], and also areas such as *intellectual disability* [32, 35] and *disability in general* [33]. The same patterns can be observed in publications from 2014 as the studies focused on *Parkinson’s disease* [42], *cognitive disability* [38, 40], and *intellectual disability* [39]. It is important to mention here that, the studies published during the year 2014, had not only discussed about disability but also *elderly* [40, 41].

Similarly, the studies published in 2015 also discussed elderly [45]. Apart from elderly, different types of disabilities had been discussed in these studies such as: *physical disability* [46, 47, 51], *disability in general* [43], *Parkinson’s disease* [49], *intellectual disability* [44, 50], *intellectual disability*, and *cognitive disability* [48]. Furthermore, the results shown a high number of occurrences of studies during the year 2016, thus, found 13 studies this year. The year 2016 is also important because only during this year, all the disabilities mentioned in Table 2, along with the concept of elderly have been discussed in the studies. For example, publications from 2016 found with focus on *physical disability* [62], *intellectual disability* [52, 54, 56], *visual disability* [63], *disability in general* [53], *cognitive disability* [57, 59–61], and *elderly* [59]. It is important to note that the majority of the studies focus on cognitive disabilities (*n* = 4). Looking at the total number of studies published over the years, it seems that this area has captured the major focus of interest in this area.

## Analysis and discussion

A general concern that is raised in the articles that were included in this study is that there exists no common definition of the terms that are used, for example, *assistive technology*. None of the articles selected for inclusion in this study discuss conceptual differences associated with the various technologies or provide definitions of terms such as *assistive technology*, *tele-rehabilitation*, or *web-based technologies*. The absence of definitions that can be used in the proper classification of the applications and services that are described in the articles makes any meaningful comparison of the various outputs of these applications and services somewhat of a challenge.

In general, the technological solutions that were suggested or reported on in the articles were aimed at (i) reducing certain limitations related to people’s disabilities *in generic terms*, and (ii) supporting increased interaction between disabled individuals and their caregivers or teachers, for example. All of the articles were consequently optimistic and assumed that disabled individuals would be able to actively participate in society if they just use the suggested technological solutions. No consideration of any potential infrastructural, socio-technical, cultural, or legal obstacles was made in any of the articles.

Only a small number of the articles included in our literature review discussed or suggested technologies that might assist individuals to become active on the labour market [33] or suggested services and applications that might support active participation [59] in social activities [44, 51], or could facilitate a disabled person’s access to educational opportunities [20, 31].

It is of further interest to note that the articles did not present any discussion of the following important issues: (i) the level of IT-literacy of the users, (ii) any possible economic restrictions associated with buying or renting the suggested services or applications or any possible complimentary help from the user’s next-of-kin or from society in general with respect to the use of suggested services, and, perhaps even more importantly, (iii) the level of disability and the user’s ability to use and adopt digital innovations. The studies were, in general, limited to the description of, or the suggestion of, prototypes [34, 63] or examples of technologies that can support some disabilities [38–40]. We also note that no analysis of the criteria that the users have to fulfil if they are to successfully use the various technologies is present in these articles.

Furthermore, the majority of the technologies suggested in the articles were aimed at solving one single issue related to a person’s disability, and they did not discuss how these technologies might be integrated into the user’s home or work environment or whether they are compatible with other existing technologies that might already be in use.

A major issue that we identified with the articles included in this literature review is the fact that the majority of the studies merely describe or discuss early ‘proof of concept studies’ and suggest how certain technologies can be triggered in the market [36, 38, 39]. Mainstream adoptions or broad market applicability of the suggested technology-based applications and web services have not been studied empirically. None of the studies discuss the effects results of the delivery of services for the organizations in charge of supporting individuals with disabilities, or the prerequisites that these technologies have to fulfil before they are granted permission to be implemented. These prerequisites may include specification of systems and guarantees concerning the level of data security and right to privacy, for example. Other requirements may include the issuing of licences, depending on the country the technology is to be used in.

Creating a ‘digital edge’ so as to remove issues that limit the participation and integration in society of individuals with disabilities implies taking advantage of technological innovations that will create new solutions; solutions that bring together physical- and digital resources, as well as the physical- and virtual spheres of existence [64]. The main challenges that face both industries and decision-makers is how they are to (i) integrate technologies and services in everyday routines, [65] and (ii) how organizations can take advantages of technological innovations in their efforts to create and offer alternatives that support social integration. Furthermore, the generic nature of the proposals presented in the articles included in the present study reduced the suggestions that were made to the level of theory only. The absence of any analysis of the level/degree of disability suffered by individuals and of any user-differences related to gender, age, culture, socio-economic position [66] and differences between various social insurance systems made the adoption of the suggested technologies and services a problematic venture. In future studies, it will be necessary to (i) define technical terms clearly, (ii) describe the goals associated with each technology properly, and (iii) analyse the proposed technological solutions in the light of existing policies and guidelines so as to enable a more grounded discussion about the technologies that can be implemented and the manner in which they can satisfy the needs of individuals with disabilities in their efforts to engage in social integration. Social inclusion cannot be achieved only by developing technologies or virtual services for the area of health- and social care. The labour market, the educational market, and the political sphere are also factors relevant to the achievement of real, and meaningful, social inclusion for all individuals in society [67]. In the case of individuals with disabilities, these three areas should stand as important priorities for the researcher if the goals (facilitating people with disabilities to integrate into the society) stated by the European Union are to be achieved [9].

## Appendix

**Table 3:** Studies relating to ICT-based applications and web-based services to reduce activity limitation and participation restriction

Ref#	year	Study objectives	Technology	Name of application(s)/service(s)	Functionality or purpose behind the application’s design	Type of disability
[58]	2016	To offer a broad explication of ‘mobility’ and ‘participation’ constructs that can be examined using GPS data.	Global Positioning System Technology (GPS)	AccuTracking software	Tracks user-mobility and participation and assists users in community mobility and participation.	*cognitive disability*
[43]	2015	To explore the potential for using mainstream ICTs as AT in lower-income countries, keeping in mind current ICT trends, the characteristics of the post-PC era, and ICT-based AT in higher-income countries.	Mobile Technology, Assistive Technology	SMS	Contact caregiver and allow for the caregiver to contact the individual.	*disability in general*
[52]	2016	To propose an intervention using a new software product and workflow for video captioning which can be used to assist student/teacher with disability in learning and teaching.	Screen Reader Technology, Assistive Technology	Universal Video Captioning Platform (UVC)	Provides a semi-automatic approach to synchronize the video captioning into accessible STEM-related videos for video lectures.	*intellectual disability*
[60]	2016	To conduct a systematic review focusing both on standard treatment methods and on innovating rehabilitation techniques used to promote upper extremity motor function in stroke patients.	Rehabilitation Technology	Virtual reality systems, virtual games, music supported therapy service	Services are used for stroke rehabilitation.	*cognitive disability*
[53]	2016	To determine the level of adoption and use of mobile technology by the participants over the timeframe of the pilot project and to assess the effect on the participants’ social integration and participation.	Mobile Technology, Assistive Technology	Instant messaging, GPS-enabled navigation with speech directions and landmark identification, currency scans and barcodes reading, training programs, mobile apps	Used to make individuals less dependent on others.	*disability in general*
[61]	2016	To evaluate the feasibility, efficacy, and safety of the JRS WAVE for use in an Australian stroke inpatient rehabilitation context.	Digital Rehabilitation Technology using Video Technology	JRS WAVE software	Helps in the rehabilitation process.	*cognitive disability*
[62]	2016	To discuss the potential for AVGs as an accessible option to increase physical activity participation for youth with physical disabilities and limitations in lower extremity function.	Game Technology	Active Video Games (AVGs)	Designed to assist in physical activity for young people with physical disabilities	*physical disability*
[68]	2016	To examine the use of a mobile technology platform, software customization, and technical support services by people with disabilities.	Mobile Technology, Assistive Technology	Mobile apps with customized options to fulfil the user’s needs and to provide 24/7 help service support for people with disabilities.	Used for empowering and ‘up-skilling’ people withdisabilities’ and increasing their levels of social participation	*disability in general*
[54]	2016	To explore a voice recognition system to support writing and reading so as to increase the user’s level of social participation.	Voice Recognition Technology, Assistive Technology	Dragon NaturallySpeakingVRS, Read+WriteGold	To assist in writing, text-to-speech software to assist reading and to develop the computer skills required to use email.	*intellectual disability*
[47]	2015	To investigate the effectiveness and safety of tele-rehabilitation intervention in pwMS for improved patient outcomes (activity limitation, participation, impairment).	Tele-rehabilitation Technology	Video conference	For reducing activity limitation and participation restriction.	*physical disability*
[48]	2015	The objective of the Singapore Tele-technology Aided Rehabilitation in Stroke (STARS) trial is to determine whether a novel tele-rehabilitation intervention for the first three months after stroke admission improves functional recovery compared to usual care.	Tele-technology, Tele-rehabilitation	Tele-therapist, Video conference	Helps in conducting face-to-face consultationswith the patient.	*cognitive disability*
[44]	2015	To investigate the effectiveness of a home-based intervention using social media to enhance social networks of young people with disabilities and communication difficulties.	Social Media, Assistive Technology	Skype, e-mail, and Facebook	Increases participation in the social community.	*intellectual disability*
[41]	2014	To summarize evidence for the effectiveness and feasibility of VR/gaming system utilisation by older adults at home for enabling physical activity with aim to improve impairments, activity limitations, or participation.	Virtual Technology	Virtual reality game system	Enables physical activity and engagement in healthy individuals and for rehabilitation.	*elderly*
[32]	2013	To increase the possibility that people with cognitive and communicative disabilities will successfully use text messaging with picture symbols and speech synthesis.	Communication Technology	Text messaging with pictures and speech synthesis	Software makes it possible to write messages with symbols. The software converts the symbols to a regular text message that can be sent to any phone. When the user receives a text message, thespeech synthesis helps the user to read the message.	*cognitive disability*
[42]	2014	To describe the past, present, and likely future applications of telemedicine for Parkinson’s disease (PD) patients.	Telecommunication Technology	Kaiser Permanente Healthcare Delivery System	Helps to access electronic medical records for their use in telemedicine, including telephone calls, secure e-mails, and video appointments, and video conferencing to facilitate virtual meetings.	*Parkinson’s disease*
[38]	2014	To investigate the perceived influence of the adoption of personal electronic response systems (‘clickers’) on undergraduate and graduate social work education by students with- and without disabilities and limited English proficiency (LEP)	Instructional Technology, Electronic Technology, Assistive Technology	Clicker Electronic Response System	To assist students in communication.	*cognitive disability*
[34]	2013	To describe the development process of a mobile Voice User Interface (VUI) for Koreanusers with dysarthria with currently available speech recognition technology by conducting a systematic user-needs analysis and by applying usability testing feedback to prototype system designs.	Mobile Technology, Speech Recognition Technology, Assistive Technology	Mobile Voice User Interface (VUI)	To improve speech recognition.	*physical disability*
[36]	2013	To examine the effectiveness of an intervention package on skill acquisition and interaction behaviour when used by two students with autism, and one student with a moderate intellectual disability.	Instructional Technology	SMART Board: Electronic Interactive Whiteboard (IWB), Notebook 10 application	Helps students to access instructional materials by using the touch screen of the SMART Board IWB with their fingers and use pens and an eraser to self-monitor task performance.	*intellectual disability*
[37]	2013	To measure the user’s willingness to share health or activity data with user’s doctor or family members and to examine concerns about privacy or security of monitoring, over one year of study participation.	Gerontechnology	Video-based Home Monitoring System	To provide care at home.	*elderly*
[33]	2013	To explore the representation/presence of disability and aging using frames, Facebook, and LinkedIn groups. Target identity/member groups on Facebook and LinkedIn were catalogued to explore the presence and representation of disability and aging identities in a socially-networked setting.	Communication Technology	Facebook and LinkedIn - online communities	Used by people with disability and elderly for community- and employment participation, not just in the specific function of activities, but as a link to larger communities of practice and professional connections.	*elderly*
[35]	2013	To understand how Technology User Groups impact operational and functional skills in IT and AT in early childhood teachers, with specific emphasis on examining the degree to which participation in a Technology User Group results in early childhood professionals developing and implementing technology-supported products in their work.	Assistive Technology	Software in each Technology Toolkit included (a) the Intellitools Classroom Suite (Cambium Learning.); (b) Boardmaker with Speaking Dynamically Pro; and (c) Writing with Symbols 2000, Microsoft Suite.	Learning tools used in the class.	*intellectual disability*
[29]	2012	To investigate ACTIV for people with stroke to improve the transition from hospital to community living.	Tele-rehabilitation Technology	Augmented Community Tele-rehabilitation Intervention (ACTIV)	ACTIV uses readily available technology, telephone and mobile phones, combined with face-to-face visits from a physiotherapist over a six-month period, to help people with stroke to resume activities they enjoyed before the stroke.	*cognitive disability*
[30]	2012	To determine whether use of Internet-based video communication for study visits improves the likelihood of participating in PD clinical trials.	Internet-based Technology	Internet-based Video Communication	For study visits to improve likelihood of participation in Parkinson’s disease (PD) clinical trials.	*Parkinson’s disease*
[26]	2012	To describe the strategies and technologies used to provide people who have moderate to severe cerebral palsy with playful and fun activities designed according to their abilities.	Assistive Technology	Gesture-based Musical Games, Virtual Musical Instrument, Audio-processing Module Application, Sound Visualization Application	To foster user participation through play and joy.	*physical disability*
[25]	2012	To discuss current research and future directions for integrated systems of technical support that include low-technology, high tech, and partner-dependent strategies for adults with severe and chronic aphasia, cognitive-communication problems resulting from traumatic brain injuries, and primary progressive aphasia.	Communication Technology, Assistive Technology	Talking Word Processing Software	The software can be used to edit the written work.	*cognitive disability*
[27]	2012	To explore female patients’ experiences of participating in a 4-week web-based home intervention after an in-house multidimensional rehabilitation program.	Web Technology	Web-based Mobile Phone	To make contact with the nurse or the patient’s carer.	*physical disability*
[31]	2012	To examine the degree to which transition-aged youths with visual impairments have used the Internet and what outcomes they have achieved following their graduation from high school.	Internet-related Technology	Email, Instant Messaging	To increase participation in the community.	*visual impairment*
[20]	2011	To describe ongoing research into the development of a specific VFT: an electronic re-creation of Mammoth Cave National Park for the Introduction to Cave and Karst Systems field course at a Midwestern research university.	Adaptive Technology	Virtual Field Trips (VFTs)	To promote equal access to undergraduate students of geo-science curricula and to enhance this academic field.	*physical disabilities*
[21]	2011	To determine the range of assistive technology items used by children with physical disabilities aged 8–18 years for participation in schoolwork.	Assistive Technology	Voice Recognition Software, Math Software, Word Prediction Software.	To help students in learning process at the compulsory school level.	*physical disabilities*
[63]	2016	To present applications of GripFab prototype software and discuss the opportunities and challenges involved in efficiently implementing 3D modelling and printing for special education use.	3D Printing Technology	GripFab, 3D Modelling Software	3D modelling offers students a powerful tool for creativity and exploration and an engaging introduction to STEM topics and can increase their participation in science and engineering.	*cognitive disability*
[55]	2016	To propose an intelligent reading support system that is capable of predicting the intended word and rendering it instead of naïvely rendering each touched word.	Voice-over Technology, Assistive Technology	“Read-What-You-Touch” systems (RWYT systems), such as word-by-word-Voiceover, and word-by-word-voice overlay, SpatialTouch Audio Annotator and Reader (STAAR), and iBook	Support Individuals with Blindness or Severe Visual Impairment (IBSVI) to discover the on-screen text by touch interaction. It renders the touched text audibly.	*visual impairment*
[56]	2016	To present a tablet-based application for activity schedules. The application addresses two domains of activity: classroom routines and verbal communication.	Assistive Technology	Tablet-based application (activity-schedule system)	To enhance the participation and learning skills in the classroom of children with autism and intellectual disabilities. The application shows the list of activities to students that they will perform in class.	*intellectual disability*
[45]	2015	To show some weaknesses in automatic speech recognition that must be addressed, as well as the need for better adaptation to the user and the environment.	Assistive Technology	e-lio application in SWEET-HOME	Provides home services to elderly people through the e-lio box (e.g., video-conferencing, calendar, photos). SWEET-Homes enables users to contact their physician through voice or tactile interface.	*elderly and visually impaired*
[49]	2015	To present a wearable system for the support of people with Parkinson’s disease (PD) and freezing of gait (FoG). The system is designed for independent use. The study also investigates the at-home acceptance of the wearable system in a study with nine PD subjects.	Wearable Technology, Healthcare Technology	Wearable System application: The system consists of three components. First, FoG episodes are detected in real time using wearable inertial sensors and a smartphone as the processing unit. Second, a feedback mechanism triggers a rhythmic auditory signal to the user to alleviate freeze episodes in an assistive mode. Third, the smartphone-based application features support for training exercises.	It enables motor training and gait assistance at home and other unsupervised environments.	*Parkinson’s disease*
[39]	2014	To evaluate the impact of introducing an augmentative and alternative communication device to a student with a co-morbid diagnosis of CP and ASD by way of a comprehensive access technology delivery protocol.	Access Technology, Assistive Technology	“GoTalk Now” application	To improve the communication skills of students.	*intellectual disability*
[22]	2011	To highlight technologies that may be useful for people with neurovascular deficits and to describe the evidence that is used to support their training and use.	Assistive Technology	Kurzweill 3000 software, Jaws and Window Eyes,	This type of software can promote improvements in attention by organizing the reading process and compiling useful information into separate documents for future review or study.	*visual impairment*
[23]	2011	To examine whether or not the use of cognitively accessible technology would improve the outcomes related to self-determination for students receiving instructions in transition planning designed to promote involvement.	Instructional Technology	WebTrek, Decision Manager, and AIMS Task Builder	A browser that provides an accessible platform to perform the most common internet tasks, such as entering the URL and searching the Internet. Task builder has been developed to enable users to create classrooms or individual tasks that are easy to import into Decision Manager. It includes images, symbols, audio prompts, and video clips.	*disability in general*
[40]	2014	To support users in their daily functioning, monitor (deviations from) patterns in daily behaviour, and to automatically detect emergency situations. and aim to inventory the end users’ needs and wishes regarding the development and design of the new integrated Rosetta system, and to describe the to-be-developed Rosetta system.	Assistive Technology	COGKNOW Day Navigator (CDN), EMERGE system	Provides support to people with dementia in the early stages of impairment in the unmet needs areas of memory, social contact, daily activities, andsafety, reminders, radio. Developed tosupport elderly people with monitoring, prevention, and emergency response by employing sensors tomonitor activities of daily living and vital data.	*cognitive disability*
[46]	2015	To gain an understanding of the experiences of clients with tetraplegia trailing assistive technologies for computer access during different stages of a public rehabilitation service.	Assistive Technology	Facebook, Skype, email, YouTube	To enhance their participation within the community.	*physical disability*
[50]	2015	To evaluate the use of visual cues and video prompting delivered by an iPad 2 with Book Creator software to teach shopping skills in the community to a young adult with Autism Spectrum Disorder and intellectual disability.	Technology	BookCreator Software	Provides visual cues and video prompting to teach shopping skills in the community.	*intellectual disability*
[51]	2015	To use IT in one’s daily activities. The purpose of the study was to obtain an in-depth perspective of how people with SCI regularly use IT to gain insight into ways IT can be used to support health and well-being in the community for a particular population.	Information Technology	Internet, Health-related websites, Social networking websites, Mobile applications, Video conferencing, E-Readers, Discussion groups, Voice transcription software, Text messaging	To increase the number of opportunities for social participation.	*physical disability*
[19]	2010	To assess the feasibility of a novel eHealth service.	Communication Technology	Improvehealth.eu	To support collaborative care management and active patient engagement, and online and phone-based care management performed by trained psychologists	*cognitive disability*
[57]	2016	A one-month randomized controlled trial with 18 stroke patients in and outpatients from two rehabilitation units: 9 performing a VR-based intervention and 9 performing conventional rehabilitation.	Virtual Technology	Reh@City	Involves memory, attention,visual-spatial abilities and executive functions tasks that are integrated to perform several daily routines.	*cognitive disability*
[28]	2012	To design a social-software environment, called RareICT, to help patients and family members in their everyday, additional un-paid work.	Web Technology	RareICT	Designed for self-care and independence in everyday living and health maintenance at the home.	*visual impairment*
[24]	2011	A summary of the legal requirements and standards that govern the presentation of health information using technology and practical guidelines for accessible health education programs.	Web Technology	Web Accessibility in Mind (Web AIM)	Provides health-related information online.	*disability in general*
[59]	2016	To report on the validity of the GPS data collected and to answer the question: *To what degree are older adults able to fulfil the requirements of a GPS assisted study?*	GPS Technology	Global Positioning System Technology	To increase their participation in the community.	*elderly*

## Footnotes

1 The International Classification of Functioning, Disability and Health (ICF), has defined disability as ‘an umbrella term for impairments, activity limitations^1^ and participation restrictions^2^, along with environmental factors^3^ that interacts with these terms’ and that restrict social inclusion.

2 Social inclusion or integration is defined “as a desired goal that required equality of opportunity and participation in the rudimentary and fundamental functions of society.” [17]
